# The First Application of Nanoelectrochemotherapy in Feline Oral Malignant Melanoma Treatment—Case Study

**DOI:** 10.3390/ani10040556

**Published:** 2020-03-26

**Authors:** Joanna Tunikowska, Agnieszka Antończyk, Nina Rembiałkowska, Łukasz Jóźwiak, Vitalij Novickij, Julita Kulbacka

**Affiliations:** 1 Department of Surgery, Faculty of Veterinary Medicine, Wroclaw University of Environmental and Life Sciences, 50-375 Wroclaw, Poland; joanna.tunikowska@upwr.edu.pl (J.T.); agnieszka.antonczyk@upwr.edu.pl (A.A.); lukasz.jozwiak@upwr.edu.pl (Ł.J.); 2 Department of Molecular and Cellular Biology, Wroclaw Medical University, 50-556 Wroclaw, Poland; nina.rembialkowska@umed.wroc.pl; 3 Institute of High Magnetic Fields, Vilnius Gediminas Technical University, 03227 Vilnius, Lithuania; vitalij.novickij@vgtu.lt

**Keywords:** feline melanoma, electrochemotherapy, nanosecond pulsed electric field, CO_2_ laser

## Abstract

**Simple Summary:**

The current study concerns feline melanoma of the oral cavity, which is rather rarely diagnosed but is usually correlated with poor prognosis. Here we proposed a new treatment modality using CO_2_ laser surgery with pulsed electric fields with ultra-short pulses in combination with bleomycin. The applied nanoelectrochemotherapy resulted in positive response and satisfactory animal recovery. Thus, nanosecond electroporation seems a reasonable anticancer approach in domestic animals.

**Abstract:**

Background: The aim of this study was to present the first domestic animal trial of nanosecond electroporation with chemotherapy and CO_2_ laser surgery. Methods: sixteen-year-old domestic cat with diagnosed melanoma on oral cavity was the case used in the study. Firstly, CO_2_ laser surgery was used for the removal of most of tumor mass. Then nanoelectrochemotherapy with bleomycin was applied including appropriate margin of healthy tissue. A 15 ± 4 kV/cm × 15 ns pulsing protocol was employed with a total of 800 ± 100 pulses. Only one session of nanoelectrochemotherapy (nanoECT) was performed. Results and conclusions: during the next two weeks areas of focal necrosis were replaced by the granulation tissue. Complete wound healing was observed four weeks after initial treatment. After 15 weeks post treatment, no local recurrence was apparent.

## 1. Introduction

Melanoma of the oral cavity is relatively rare in cats, and its diagnosis is usually associated with poor prognosis [1]. Local control of this tumor requires wide surgical excision with additional medical treatment [2].

Electroporation is a phenomenon of pulsed electric field (PEF) initiated permeabilization of biological cell plasma membrane, which serves as a basepoint of many successful biomedical and biotechnological methodologies including electrochemotherapy (ECT) for cancer treatment. ECT is as effective combination of chemotherapeutic and electroporation, where microsecond pulses up to few kV are applied. The available research studies proved that electrochemotherapy can be efficiently applied in combination with bleomycin in soft-tissue sarcomas [3] and tumors with a small surgical safety margin, such as the limb extremities, skull, oral cavity, neck and perianal region [4]. Nanoelectrochemotherapy (NanoECT) is a new modality of electrochemotherapy not applied in clinical practice, which employs nanosecond range (10–300 ns) but high intensity pulses for cell membrane reorganization and simultaneous improved drug delivery [5,6]. The previous study demonstrated that 10ns pulses supported strontium ranelate transport across cell membranes in various cancer cell models, in particular in resistant cells. It was proved that nsPEF significantly inhibited tumor cell growth and affected intracellular structure [7]. In the other experimental examination, nanosecond pulses assisted photosensitizer delivery and simultaneously enhanced the final photodynamic effect in gastric cancer, melanoma and skin cancer [8]. Nanoelectroporation was also indicated as an apoptotic signal stimulator in various human cancer cell models [9,10,11].

The clinical applications of the microsecond ECT revealed a promising usefulness in local tumor control, thus the main objective of this study was to evaluate application of nanoECT as a supportive treatment for incomplete surgical excision in local tumor control in the domestic animal.

## 2. Materials and Methods

### 2.1. Case Presentation

A 16-year-old, domestic shorthair cat was referred to the veterinary center with chronic renal failure, ptyalism, aphagia and a deformation on the left side of a maxillary area.

The oral mass was located in the caudal portion of the left maxilla, involving caudal lip margin, buccal and gingival tissues. Left mandibular lymph node was firm and fixated in comparison to the opposite site. Based on abdominal ultrasound examination and thoracic radiographs, no distant metastases at initial evaluation were noted. Fine needle aspiration of the tumor mass and left submandibular lymph node revealed infiltration with melanocytes and melanoblasts indicating malignant melanoma. The cat was staged III according to the alternative system for staging canine oral melanoma [12]. The owner of the cat chose and approved the palliative treatment in form of nanoelectrochemotherapy.

### 2.2. CO_2_ Laser Surgery and nanoECT Treatment

**Figure 1:**
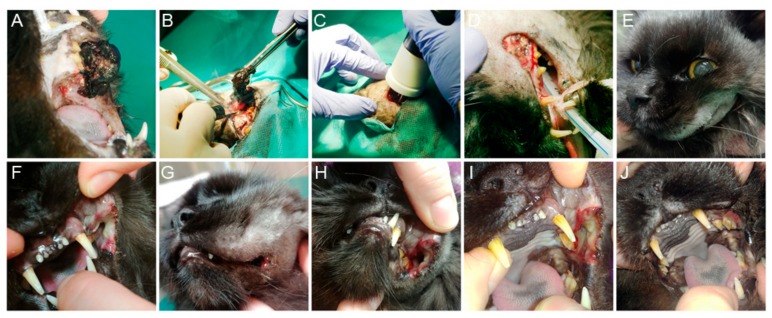
Case representation before, during and after nanoelectrochemotherapy (nanoECT): (**A**) malignant melanoma of oral cavity in cat prior treatment; (**B**) marginal excision with CO_2_ laser; (**C**) nanoECT after tumor resection; (**D**) oral cavity after tumor excision and nanoECT; (**E**) swelling of the upper lip and cheek after nanoECT; (**F**) tissue necrosis 48 h after nanoECT; (**G**) slight swelling of the upper lip 4 days after nanoECT; (**H**) focal necrosis with areas of tissue healing 5 days after nanoECT; (**I**) focal necrosis with areas of tissue healing 10 days after nanoECT; (**J**) oral cavity in cat 6 weeks after nanoECT. (own photos)

Surgery was conducted under short infusion anesthesia and local anesthesia (Figure 1A,B). The cat was given dexmedetomidine (Dexdomitor, Orion Pharma, Espoo, Finland) at a dose of 40 mcg/kg intramuscularly in premedication. General anesthesia was induced with propofol (Propofol-Lipuro 1%, B. Braun Melsungen AG, Melsungen, Germany) to effect. The cat was intubated with a cuffed endotracheal tube (internal diameter 3.5 mm) and was breathing spontaneously with oxygen-enriched air (Figure 1D). Following maxillary, nerve block was performed using lidocaine (Lignocainum Hydrochloricum WZF 2%, Polfa Warsaw, Poland). Additional propofol was administered as a bolus of 1–2 mg/kg when needed.

**Figure 2:**
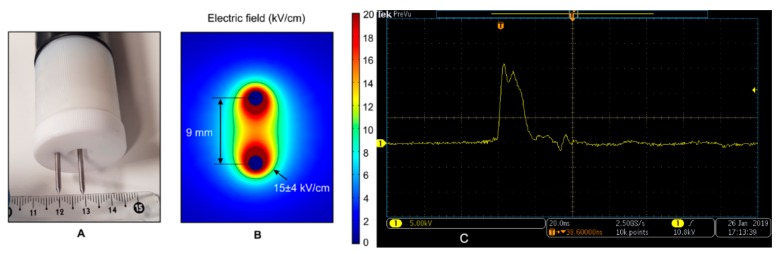
The representation of specialized electrode and its characteristic: (**A**) Two-needle electrode and (**B**) electric field distribution; (**C**) nanosecond pulse shape delivered (15 kV/cm × 15ns, 800 ± 100 pulses).

The tumor mass was excised marginally from the gingival and buccal area with a CO_2_ laser (Eraser C, Seoul, Korea) working in continuous mode and 10 W of power output (Figure 1B). The excisional site was injected with bleomycin (Bleocel, Celon Laboratories Ltd., Telangana, India) at the dose of 2 mg/m^3^ and nanoelectroporated (Figure 1C). For pulse delivery, the PPG-20 generator (FID Technology, Burbach, Germany) and an applicator with needle-type (9 mm gap) steel electrodes were used. A 15 ± 4 kV/cm × 15 ns pulsing protocol was employed and a total of 800 ± 100 pulses were delivered to the site at repetition frequency of 200 Hz. (Figure 2A–C). The draining nodes were not incorporated in the electroporation field.

The patient received buprenorphine (Bupaq Multidose 0.3 mg/mL Richter Pharma AG, Feldgasse, Austria) at a dose of 20 mcg/kg every 8 hours for the first 3 days for postoperative pain management. Amoxicillin with clavulanic acid (Synulox 50 mg, Pfizer Trading Poland, Warsaw, Poland) at a dose of 12.5 mg/kg was administered every 12 hours for two weeks. Single injection of a dexamethasone (Rapidexon 2 mL/mL, Eurovet Animal Health B.V., Bladel, Netherlands) at a dose of 1 mg/kg was given to reduce massive swelling of the upper lip and cheek that occurred at the second day after surgery.

### 2.3. Post Treatment Observations

Three days after the surgery, appetite restoration and normal food intake was noticed. During the next two weeks areas of focal necrosis were replaced by the granulation tissue (Figure 1I). Complete wound healing was observed 4 weeks after initial treatment (and 6 weeks in Figure 1J). Although 15 weeks after ECT no local recurrence was evident, progression of submandibular lymph node enlargement enabled food intake Because no visible distant metastases in abdominal or thoracic cavity were found, decision on surgical lymph node excision was made. Malignant melanoma of the lymph node was confirmed in histopathological examination. Two weeks after lymphadenectomy, worsening of the general health status was observed. Abdominal ultrasound revealed nodular metastatic spread into the visceral organs and euthanasia of the patient took place.

Intraoperatively, no negative tissue effects during nanoelectrochemotherapy (nanoECT) were observed as well as no involuntary skeletal muscle contractions that are characteristic of electrochemotherapy. Despite the promising treatment effects during the perioperative period, further observation of the long-term effects of nanoECT is necessary.

## 3. Discussion

Oral melanoma is a treatment-resistant cancer in both human and veterinary patients that requires multimodality approaches. Local recurrences of oral malignant melanoma commonly undergo excisions on multiple occasions until the patients die due to metastatic disease [13]. The retrospective study on canine oral melanoma has not found any clear survival benefit with any systemic adjuvant therapy, involving chemotherapy or vaccines against melanoma [14]. The search for the supportive treatment that may improve local tumor control and restore life quality seems to still be a problem in oral malignant melanoma treatment. 

In our patient, due to the advanced stage of the disease the intent of the treatment was to obtain prolonged local control without causing massive surgical trauma. We decided to debulk the main tumor mass with CO_2_ laser because of its hemostatic properties and relatively small thermal effect on surrounding tissues, also no suture placement was necessary. Beneficial in the surgical treatment with CO_2_ laser in oral cavity pathologies is also the reduction of postoperative pain and reduced scarring [15,16].

Because our patient was suffering from chronic renal failure, supportive treatment like radiation therapy was rejected due to the necessity for multiple sessions and patient anesthetic sedation. The recent studies indicate that irreversible electroporation (IRE) or ECT protocols were extended to nanosecond pulses range duration [17]. Protocols involving nsPEF in vivo application carries the risk of the increased heating in the place of treatment. However, Nuccitelli et al. used 30 kV/cm × 100 ns × 2000 pulses and it was highlighted that up to seven pulses/second can be used without significantly raising the tumor temperature [18]. In our case, we limited the total pulse energy to be on the safe side without measuring the current. The parameters were super-positioned to available in vivo melanoma studies in mice, which is an acceptable approximation in terms of conductivity. We used 200 Hz frequency (200 pulses/second), but the total energy of the burst was more than 60-fold lower, which allowed us to ensure a nonthermal treatment. In the other studies authors were able to maintain constant temperature (43 °C) during the nanosecond procedure [19]. Currently nsPEF clinical trials are not widespread. They are mostly used on melanoma or skin cancers. There was performed split-dose treatments on (KLN 205) squamous carcinoma using 300 ns pulses with bleomycin. Similar electroporation parameters (300 ns) in contrast to ms range were used against murine melanoma. Authors observed that nsPEF reduced or delayed effects on green fluorescent protein (GFP) quenching but it had stronger and more significant effects in tumor size reduction [20]. Garon et al. performed a single in vivo case study using 20 ns electric pulses demonstrating pancreatic tumor regression [21]. One of the major findings is that nanosecond electroporation can permeabilize external and internal cell membranes and distribute the drug more efficiently [22]. Additionally, the application of short electrical pulses is more beneficial due to the minimization of muscle contraction. In the case of animals, treatment of this issue is relevant to conducting anesthesia procedures, which are performed to keep the treated animal in light anesthesia.

## 4. Conclusions

We have presented the effect of PEF in the submicrosecond range applied to the melanoma case detected in domestic cat. The nsPEF combined with local bleomycin injection resulted in promising treatment effects in this palliative case during the perioperative period. However, this new methodology requires further observation of the long-term effects of nanoelectrochemotherapy.
